# Perioperative Outcomes of Nephrectomy for Autosomal Dominant Polycystic Kidney Disease

**DOI:** 10.1097/UPJ.0000000000000921

**Published:** 2025-12-17

**Authors:** Michael Waseer Bacchus, Vivian Wong, Akshay Sood, Eric A. Singer, Shawn Dason

**Affiliations:** 1 Division of Urologic Oncology, The Ohio State University Comprehensive Cancer Center, Columbus, Ohio

**Keywords:** polycystic kidney, nephrectomy, perioperative outcomes

## Abstract

**Introduction:**

Patients with autosomal dominant polycystic kidney disease (ADPKD) frequently require nephrectomy before renal transplant or for clinical symptoms. We encountered no population-based data on perioperative outcomes for nephrectomy in patients with ADPKD, indicating a knowledge gap for patient counseling and quality benchmarking.

**Methods:**

We analyzed the American College of Surgeons National Surgical Quality Improvement Program database (2015-2022) to identify patients undergoing nephrectomy with a diagnosis of ADPKD. The primary outcome was major complications. Multivariable logistic regression was used to identify predictors of outcomes.

**Results:**

The cohort comprised 823 patients with a median age of 54 years (range 19-87). Preoperative hypertension (79.3%), dialysis (49.2%), and steroid use (44.8%) were common. Major complications occurred in 7.0% of patients. Minimally invasive surgery was associated with lower major complication risk (odds ratio 0.269; *P* < .001), reduced length of stay (3 vs 6 days), and decreased transfusion rates (7.8% vs 29.1%). Preoperative steroid use was also associated with reduced risk. Dialysis status and bilateral nephrectomy were not significant predictors. Thirty-day mortality was noted in 4 patients (0.5%).

**Conclusions:**

Major complications and death are rare after nephrectomy for ADPKD despite the high rate of dialysis and renal transplantation in this population. When technically feasible, minimally invasive surgery may be beneficial in nephrectomy for ADPKD.

Autosomal dominant polycystic kidney disease (ADPKD) is the most common cause of inherited chronic kidney disease, with an estimated incidence of 1/1000 annually.^1^ Responsible for 5% to 10% of all cases of end-stage renal disease within the United States, ADPKD is characterized by the development of multiple large cysts within the patient’s kidneys, which diminish renal function and impact extrarenal abdominal structures through mass effect.^2,3^ Symptom onset typically occurs in the early thirties and often presents with various combinations of hematuria, flank pain, palpable abdominal masses, and downstream effects of arterial hypertension.^4^ Medical management of ADPKD and associated renal dysfunction includes angiotensin-converting enzyme inhibitors, vasopressin antagonists (ie, tolvaptan), and fluid optimization to treat hypertension and minimize end-stage renal disease progression.^5,6^ Unfortunately, ADPKD commonly results in end-stage renal disease requiring dialysis or kidney transplantation.^5,6^

**UPJ Insight**

**Study Need and Importance**
Patients with autosomal dominant polycystic kidney disease (ADPKD) often require nephrectomy to facilitate renal transplantation or because of clinical symptoms. Despite this, perioperative outcomes in this population remain poorly characterized at a national level and represent a knowledge gap. Prior studies are limited by single-institution designs and small sample sizes, limiting evidence-based counseling. This study used the American College of Surgeons National Surgical Quality Improvement Program (NSQIP) to assess perioperative outcomes after unilateral or bilateral nephrectomy in patients with ADPKD.
**What We Found**
The cohort comprised 823 patients with a median age of 54 years (range 19-87). Preoperative hypertension (79.3%), dialysis (49.2%), and steroid use (44.8%) were common. Major complications occurred in 7.0% of patients. Minimally invasive surgery was associated with lower major complication risk (odds ratio 0.269; *P* < .001), reduced length of stay (3 vs 6 days), and decreased transfusion rates (7.8% vs 29.1%). Preoperative steroid use was also associated with reduced risk. Dialysis status and bilateral nephrectomy were not significant predictors. Thirty-day mortality was noted in 4 patients (0.5%).
**Limitations**
As an NSQIP-based study, these findings reflect data from participating hospitals only and lack ADPKD-specific variables such as kidney size, transplant timing, or nephrectomy indication. The database also lacks Clavien-Dindo grading, limiting complication stratification. Furthermore, this database does not differentiate between laparoscopic, robotic, or hand-assisted minimally invasive surgery techniques.
**Interpretation for Patient Care**
Major complications and death are rare after nephrectomy for ADPKD, despite the rate of preoperative dialysis and renal transplant in this population. When technically feasible, minimally invasive approaches should be prioritized for reduced morbidity and faster recovery. The results from this study help support risk counseling, surgical planning, and shared decision-making in patients with ADPKD considering nephrectomy.

Many patients with ADPKD require removal of one or both of their kidneys for size-related symptoms or to make space for a transplant kidney.^5,6^ Despite this, perioperative outcomes of individuals with ADPKD undergoing nephrectomy are not well characterized. This creates a challenge for patient counseling and quality benchmarking. To address this need, we analyzed the American College of Surgeons (ACS) National Surgical Quality Improvement Program (NSQIP) database to assess perioperative outcomes after unilateral or bilateral nephrectomy in patients with ADPKD.

## Methods

### Data Source

This study was exempt from our Institutional Review Board review. This analysis used the ACS-NSQIP database. This nationally validated database records key preoperative and 30-day risk-adjusted postoperative outcomes associated with surgical procedures. To promote quality assurance, the database is maintained and audited through trained surgical clinical reviewers associated with each ACS-NSQIP participating hospital. For this study, we obtained and reviewed the NSQIP Participant Use Data files for the years 2016 through 2022, inclusive.

### Study Population

Using NSQIP data from 2015 through 2022, we identified adult patients (older than 18 years) undergoing surgical cases that contained current procedural terminology (CPT) codes consistent with nephrectomy (CPT 50220, 50230, 50234, 50545, 50546, 50548, 50549) and concurrent ICD-10 codes consistent with the diagnosis of ADPKD (Q61.2).

### Variables

Patient demographics collected included sex (male/female), age (years), height (inches), weight (pounds), body mass index (pounds divided by inches squared), current smoking status (yes/no), and current functional status (categorized as independent, partially dependent, dependent). Other discrete data associated with comorbidities included history of diabetes (including if insulin or noninsulin dependent), history of chronic obstructive pulmonary disease (COPD), and history of congestive heart failure. The use of dialysis for end-stage renal disease and steroids (which can be associated with renal transplant status) before operation was also identified. Finally, preoperative anemia (hematocrit as a continuous variable) and preoperative pancytopenia (categorized as a discrete variable of either present or absent) were noted for each patient. Variables associated with the operation included minimally invasive vs open surgery and surgeon specialty (urology vs general surgery).

### Outcomes

Our primary outcome was major complication rate, defined in this project as a composite outcome that included death, cardiac arrest, myocardial infarction, pulmonary embolism, cerebrovascular accident, septic shock, reoperation, reintubation, and renal failure requiring dialysis. Secondary outcomes included length of stay, transfusion rate, system-specific complications, operative time, readmissions, reoperations, or reintubations.

### Statistical Analysis

Baseline patient demographics were assessed for differences with the Student’s *t* test (continuous variables) or χ^2^ test (categorical variables). Logistic regression was used to assess the role of predictors on the primary and secondary outcomes. Predictors selected for multivariable analysis included those significant on univariable analysis or those that had clinical relevance. Collectively, this included surgical approach, smoking status, COPD, dialysis, steroid use, and anemia. Statistical analysis was conducted with IBM SPSS version 21 (IBM Corporation, Armonk, NY).

## Results

### Patient Demographics

In total, 823 patients met the criteria for inclusion in our study. Baseline characteristics are described in Table 1. The median age of patients was 54 years (range 19-87). The average BMI was 28.5 kg/m^2^ (IQR: 24.9-32.8). The comorbidity profile was as expected for this population, with 44.8% having a baseline preoperative steroid use and 49.2% being on dialysis preoperatively.

**Table 1.** Preoperative Characteristics and Intraoperative Outcomes of Patients Undergoing Nephrectomy for Autosomal Dominant Polycystic Kidney Disease

Descriptive characteristic	Cohort
Total patients, No.	823
Age, median (range), y	54 (19-87)
BMI, median (range), kg/m^2^	28.5 (15.8-53.5)
Hypertension, No. (%)	652 (79.3)
Dialysis, No. (%)	405 (49.2)
Steroid use, No. (%)	369 (44.8)
Preoperative anemia, No. (%)	251 (30.5)
Congestive heart failure, No. (%)	19 (2.3)
Functionally independent, No. (%)	812 (98.7)
Current smoker, No. (%)	80 (9.7)
Operative time, median (IQR), min	182 (137-236)
Minimally invasive surgery, No. (%)	344 (41.8)
Bilateral nephrectomy, No. (%)	217 (26.4)
Concurrent hernia repair, No. (%)	37 (4.5)

### Operative Characteristics

Table 1 includes the operative characteristics of this cohort. The median time of operation was 182 minutes (IQR: 137-236 minutes); 69.4% of cases were performed by urologists, and 29.8% were performed by general surgeons.

Of the cases, 41.8% were performed as minimally invasive surgery (MIS). Urologists were more likely to perform MIS than general surgeons (47% vs 31% of nephrectomies, *P* < .01). The median time for MIS nephrectomy was 182 minutes, which did not differ significantly from open surgery and did not differ by specialty.

Collectively, 26.4% of cases included bilateral nephrectomy. When bilateral procedures were performed, 23.7% were MIS and 76.3% were open. General surgeons were more likely to perform bilateral nephrectomy than urologists (34.7% vs 21.4%, *P* < .01). The median operative time for MIS bilateral nephrectomy was 232 minutes (IQR 158-309) vs 182 minutes for open (IQR 133-228, *P* < .01); 4.5% of overall cases included concurrent hernia repair.

### Postoperative Outcomes

Postoperative outcomes are summarized in Table 2. The median length of stay was 4 days (IQR: 3-7). Seven percent of patients had a major complication and 25% had any complication. Among the major complications, infectious complications were the most common. On the day of the operation (including intraoperatively), 11.4% of patients received a blood transfusion and 5.0% of patients received a transfusion on subsequent days after surgery. After discharge, 11.3% of patients were readmitted to the hospital within 30 days; 3.4% of patients required reoperation within 30 days. Thirty-day mortality was noted in 4 patients (0.5% of total cases).

**Table 2.** Postoperative Complications Within 30 Days of Patients With Autosomal Dominant Polycystic Kidney Disease Undergoing Nephrectomy

Postoperative outcome	Cohort
Major complications, all, No. (%)	58 (7)
All complications, No. (%)	206 (25)
Readmission, No. (%)	93 (11.3)
Reoperation, No. (%)	28 (3.4)
Mortality, No. (%)	4 (0.5)
Infectious complications, No. (%)	82 (10.0)
Wound complications, No. (%)	19 (2.3)
Pulmonary complications, No. (%)	30 (3.6)
Cardiac complications, No. (%)	12 (1.5)
Venous thromboembolism, No. (%)	11 (1.3)
New dialysis requirement, No. (%)	20 (2.4)
Length of stay, mean (IQR), d	4 (3-7)

### Predictors of Complications

The use of MIS and preoperative steroid use (which can be associated with renal transplant status) was associated with lower rates of major complications and overall complication rates (Table 2). Patients undergoing MIS also had a statistically significant reduced length of stay (3 days MIS vs 6 days open, *P* < .05) and decreased need for blood transfusion (7.8% MIS vs 29.1% open, *P* < .01).

On univariable logistic regression, MIS, steroid use, normal preoperative hematocrit, nonsmoker status, and lack of COPD were associated with reduced major complication risk on logistic regression. In our multivariable analysis, MIS, nonsmoker status, and lack of COPD were associated with reduced major complication risk. These results are reflected in Tables 3 and 4, and the Figure.

**Table 3.** Association Between Prognostic Factors and Percentage of Patients in Our Population With Major Complications and Any Complications

Variable	Major complication, %	Any complication, %
Minimally invasive surgery	2.9	13.1
Open surgery	10.0	33.6
Preoperative steroid use	4.6	19.0
No preoperative steroid use	9.0	30.0

**Table 4.** Univariate and Multivariate Logistic Regression Analysis of Associations Between Prognostic Factors With Major Complication Rate

Variables	Univariate analysis		Multivariate analysis	
	OR (95% CI)	*P* value	OR (95% CI)	*P* value
Minimally invasive surgery	0.269 (0.134-0.539)	< .001	0.269 (0.127-0.573)	< .001
Smoker	3.370 (1.755-6.469)	< .001	3.085 (1.501-6.341)	.002
COPD	3.342 (1.212-9.216)	.02	3.937 (1.295-11.965)	.016
Dialysis	1.504 (0.875-2.585)	.139	0.548 (0.249-1.209)	.136
Steroid use	0.486 (0.272-0.871)	.015	0.441 (0.188-1.030)	.059
Anemia	1.784 (1.010-3.152)	.046	1.468 (0.787-2.737)	.228
Age	1.010 (0.982-1.039)	.467		
Urologic surgeon	1.287 (0.701-2.362)	.416		
BMI	0.992 (0.946-1.040)	.724		
Diabetes	0.789 (0.370-2.127)	.789		
Hypertensive medication use	0.689 (0.331-1.432)	.319		
Bilateral procedure	0.972 (0.529-1.788)	.928		
Independent functional status	1.326 (0.708-2.485)	.378		

Abbreviations: COPD, chronic obstructive pulmonary disease; OR, odds ratio.

**Figure.**
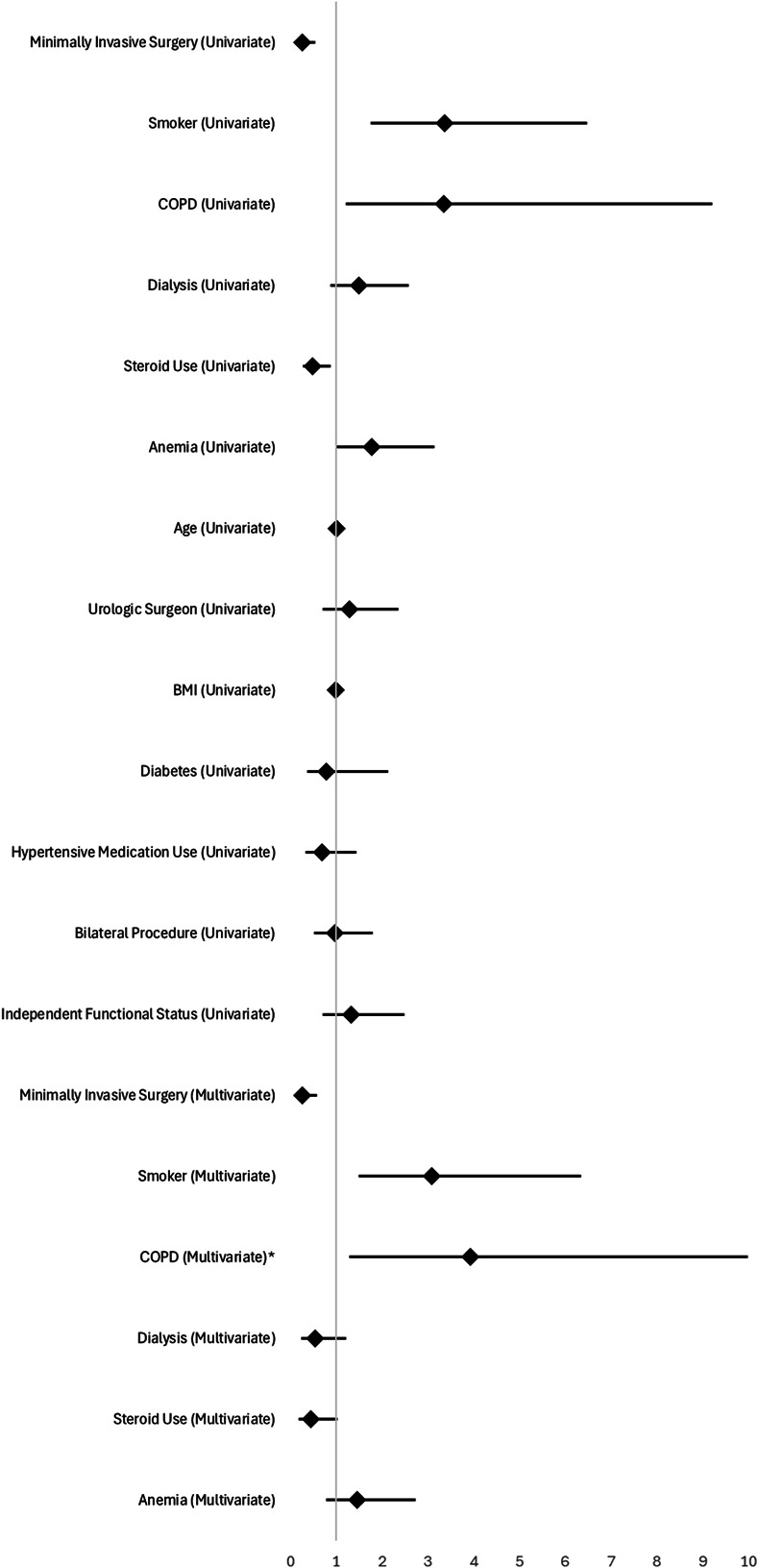
Forest plot of univariate and multivariate logistic regression analysis of associations between prognostic factors with major complication rate. *Chronic obstructive pulmonary disease (COPD; multivariate) has an upper bound of 15.902, which extends beyond the maximal axis of 10 for visual clarity.

## Discussion

In this study, we performed a comprehensive retrospective review of patients undergoing nephrectomy for ADPKD using the ACS-NSQIP database. Hypertension, preoperative dialysis, and preoperative steroid use were unsurprisingly more common than typically seen in a cohort with a median age of 54. Most patients underwent open surgery and unilateral nephrectomy, although MIS (41.8%) and bilateral nephrectomy (26.4%) were relatively common. On multivariable logistic regression, undergoing MIS, nonsmoker status, and lack of COPD had a statistically significant association with decreased major complication rate.

ADPKD is a common pathology, and further research is warranted to better characterize the needs, characteristics, and complications of patients who require nephrectomy. Prior studies have discussed the indications for nephrectomy within this patient population, which overwhelmingly include pain, recurrent urinary tract infections, urolithiasis, and hematuria.^6^ There have been prior studies that have looked at the complications and outcomes of patients undergoing nephrectomy (both prerenal and postrenal transplant), but many are limited by being single-institution studies and smaller sample sizes.^7,8^ Our study sought to obtain population-level data and analysis and had 2 primary objectives. First, we sought to identify the most common complications associated with patients with ADPKD undergoing nephrectomy. Second, we performed multivariable analysis to identify preoperative or operative characteristics that could be prognostic factors for postoperative outcomes. In turn, clinicians could use these factors to stratify patients by complication risk, educate patients about complications before surgery, and guide medical comorbidity optimization before surgery.

Understandably, the most common comorbidities that were seen among the studied cohort included hypertension, history of dialysis (as a proxy for end-stage renal disease), and history of steroid use. These results correspond with demographics reported previously in the literature.^9,10^ Our demographic data collection did not include patient information regarding sex or race, but prior studies have not shown a sex preference for ADPKD.^11^ Within our subgroup of patients with major postoperative complications (7% of total patients), infection, such as sepsis or septic shock, was the most frequent major complication (10% of the subgroup). While infection has been reported in other ADPKD nephrectomy studies, infection being the plurality of complications is a new finding.^12^ It is unclear why rates of infection were higher within our study, but this may reflect an overall higher comorbidity profile as indicated by the rate of patients on dialysis and steroids preoperatively, a high open surgery rate (58%), a high bilateral procedure rate (26%), and improved capture of sepsis readmissions through NSQIP when compared with single-institution reports. Compared with previous studies examining complications after nephrectomy using NSQIP, our rate of infections-specific major complications is higher (10% of our cohort vs 1%-6% in other studies based on a technique used).^13,14^ Our study also provides context for the frequency of common complications such as cardiac and pulmonary etiologies that have been identified and discussed in other studies. Compared with similar studies, our observed rates of cardiac complications and pulmonary complications are comparable with prior nephrectomy series.^15,16^

MIS was associated with a reduced major complication rate, length of stay, and need for blood transfusion. This affirms previously reported literature.^17-19^ Of note, MIS cases in general may not be feasible for all patients depending on the size of the polycystic kidney(s) and other anatomical considerations. Patients may also be more likely to undergo MIS if they have smaller kidneys or are undergoing unilateral nephrectomy compared with bilateral nephrectomy, which may also influence these results. Nonetheless, these results support preferencing MIS when technically feasible.

Steroid use (which can be related to renal transplant status) was also associated with lower major complication risk. This was statistically significant on univariable but not multivariable analysis (multivariable odds ratio 0.441 [95% CI 0.188-1.030], *P* = .059). In prior studies comparing patients who received nephrectomy before vs after transplant, it has been demonstrated that patients receiving nephrectomy after transplant were more likely to have shorter hospital stays and no postoperative kidney failure.^7^ In the study above, complication rates were also lower when nephrectomy was performed post transplant compared with prior; however, this finding was not statistically significant.

One of this study's strengths is the data set's composition. ACS-NSQIP is a multi-institutional dataset that allows for a large sample size while minimizing source bias seen in single-institution studies. In addition, quality assurance is maintained through auditing by surgical clinical reviewers who have been trained explicitly for NSQIP database maintenance. Together, these factors help strengthen the quality of the downstream analysis. Furthermore, NSQIP catalogs information about patients for 30 days after surgery and not just the encounter from their nephrectomy; as a result, this also includes readmission data that further clarifies a given patient’s clinical course in the perioperative period. Overall, ACS-NSQIP provides a high-quality view of these patients at a large enough sample size that allows these findings to be substantial and improve its generalizability.

There are limitations to this study, however, and those should be acknowledged. First, the patient population reviewed within this study is derived directly from the ACS-NSQIP database, which is formed through submissions from ACS-NSQIP participating hospitals. Therefore, patient cases from hospitals not affiliated with ACS-NSQIP are not represented within this study, which affects the generalizability of this work. In addition, the ACS-NSQIP database captures procedures from various surgical cases and is not explicitly designed for nephrectomies (or urologic cases in general). Therefore, specific demographics such as the size of kidneys, years on dialysis, or time relation to any possible transplants could not be obtained. In addition, while we are able to collect procedure and diagnosis codes from NSQIP, clarity on true surgical indication is absent in our study. For this reason, we cannot differentiate patients who underwent nephrectomy for ADPKD for indications such as preparation for transplant vs recurrent urinary tract infection or recurrent bleeding. Furthermore, our analysis also did not differentiate laparoscopic vs robotic cases. Given that our study found statistically significant differences in outcomes such as length of stay, certain complications, and transfusion requirements, the inability to further stratify these findings between robotic vs laparoscopic vs laparoscopic hand-assisted cases is an additional limitation. Another limitation to our study is the lack of granularity with certain variables, such as the degree of hypertension (treated as a binary in our research). Further studies that use a nephrectomy-specific database that captures these variables would be beneficial in elucidating the impact of these characteristics on patient outcomes and the potential to optimize patients before surgery. Finally, the ACS-NSQIP database does not classify patients using the Clavien-Dindo system to grade the severity of complications objectively and reproducibly. For this reason, we approximated key complications by using well-defined events within the NSQIP system as defined in our methodology. The use of a composite complication outcome as used in our study has also been seen in other NSQIP-based studies.^13,14^ Regardless, the inability to use the Clavien-Dindo system, which has been established as a standard for reporting surgical complications, is a key limitation that should also be addressed.

## Conclusions

Major complications and death are rare after nephrectomy for ADPKD despite the rate of preoperative dialysis and renal transplant in this population. When technically feasible, MIS is likely beneficial in nephrectomy for ADPKD.
